# Spatio-temporal patterns and determinants of measles incidence in Ethiopia between 2018 and 2024

**DOI:** 10.3389/fpubh.2026.1760450

**Published:** 2026-04-09

**Authors:** Habtamu Milkias Wolde, Ali Raza, Wakjira Kebede, Beom-Seok Jeong, Yeongjae Kim, Sunyeup Kim, Jae June Dong, Youngoh Bae, Seung Won Lee

**Affiliations:** 1 Department of Precision Medicine, Sungkyunkwan University School of Medicine, Suwon, Republic of Korea; 2 School of Medical Laboratory Sciences, Jimma University, Jimma, Oromia, Ethiopia; 3 Department of Artificial Intelligence, Sungkyunkwan University, Suwon, Republic of Korea; 4 Department of Family Medicine, Kangbuk Samsung Hospital, Sungkyunkwan University School of Medicine, Seoul, Republic of Korea; 5 Department of Neurosurgery, Korean Armed Forces Capital Hospital, Seongnam, Republic of Korea; 6 Department of Metabiohealth, Sungkyunkwan University, Suwon, Republic of Korea; 7 Personalized Cancer Immunotherapy Research Center, Sungkyunkwan University School of Medicine, Suwon, Republic of Korea; 8 Department of Medical Artificial Intelligence, Sungkyunkwan University, School of Medicine, Suwon, Republic of Korea

**Keywords:** Ethiopia, hotspots, measles, spatial lags, spatio-temporal modeling, temporal lags, vaccination

## Abstract

**Background:**

Despite the availability of an effective vaccine, measles remains a major public health concern in Ethiopia, with recurrent outbreaks and substantial spatial heterogeneity. Understanding its spatio-temporal patterns and determinants is critical for optimizing control strategies and achieving elimination goals.

**Methods:**

A retrospective spatio-temporal analysis was conducted using national measles surveillance data from 2018–2024, aggregated at the zonal level. Geographic clustering was assessed using Moran's I, Getis-Ord Gi^*^, and Local Indicators of Spatial Association (LISA) statistics. A negative binomial regression model incorporating spatial and temporal effects was fitted to identify determinants of measles distribution, integrating epidemiological, environmental, nutritional, and socioeconomic variables.

**Results:**

Between 2018 and 2024, 71,635 measles cases were reported, with the highest burdens observed in Oromia, Somali, Southern Ethiopia, and parts of Amhara. Significant spatial clustering was detected (Moran's I = 0.154, *p* = 0.003), with persistent hotspots in southern and southwestern zones. The model showed that higher night-light intensity (IRR = 2.21, *p* < 0.001) and temporal (IRR = 1.24, *p* = 0.028) and spatial lag effects (IRR = 1.73, *p* < 0.001) were strongly associated with increased measles incidence. Higher temperature (IRR = 0.78, *p* = 0.005) and relative wealth index (IRR = 0.40, *p* < 0.001) were inversely associated, while underweight prevalence and distance to health facilities were not significant predictors of measles distribution.

**Conclusion:**

Measles transmission in Ethiopia exhibits clear spatial clustering and temporal persistence, strongly influenced by socioeconomic inequities, human concentration, and climatic conditions. Incorporating spatio-temporal modeling into routine surveillance can enhance early detection and guide geographically targeted immunization, nutrition, and equity-focused interventions toward measles elimination.

## Introduction

Measles is a highly contagious viral disease caused by *Measles morbillivirus* which is a member of the *Paramyxoviridae* family. Transmission occurs through respiratory droplets or direct contact with nasal and throat secretions of infected individuals, with the virus capable of remaining airborne or viable on surfaces for up to 2 h (1, 2). The disease typically presents with high fever, cough, coryza, conjunctivitis, and a maculopapular rash that begins on the face and spreads to the trunk and limbs. Complications such as pneumonia, encephalitis, and severe diarrhea contribute to significant morbidity and mortality, particularly among malnourished children and those with weakened immune systems. Globally, measles caused an estimated 136,000 deaths in 2022, mostly among unvaccinated children under 5 years of age (3). Although no specific antiviral therapy exists, measles is preventable through vaccination, with the measles-containing vaccine (MCV) providing approximately 93% and 97% protection after the first (MCV_1_) and second (MCV_2_) doses, respectively (4).

Despite the availability of an effective and inexpensive vaccine, measles remains one of the leading causes of vaccine-preventable child mortality worldwide (5). In 2023, there were approximately 10.3 million infections globally (3), with more than 130,000 deaths reported in 2022 representing a 43% increase in cases compared with 2021 (6). The World Health Assembly set targets in 2010 to reduce global measles incidence to fewer than five cases per million population and mortality by 95% by 2015 (7). Although many regions have achieved substantial progress, several African countries, including Ethiopia, continue to experience recurrent outbreaks despite long-standing vaccination efforts and adoption of the regional goal of measles elimination.

In Ethiopia, measles remains a major public health problem. Between 2021 and 2023, more than 15,000 confirmed cases were reported, with a case fatality ratio of 1.1% (8). According to WHO and UNICEF estimates, MCV_1_ and MCV_2_ coverage in 2021 were 54% and 46%, respectively far below the recommended levels required for herd immunity (9). The country experiences recurrent outbreaks, particularly in Oromia, Southern, and South-Western regions, which consistently report the highest annual case numbers (10). Factors contributing to these persistent outbreaks include low vaccination coverage, delayed or incomplete supplementary immunization activities (SIAs), and immunity gaps due to population displacement, malnutrition, and service disruptions during conflict or natural disasters.

Recent studies have increasingly applied spatial and temporal analytical approaches to better understand the transmission dynamics of measles and to identify geographic areas at elevated risk of outbreaks. Spatial analyses have demonstrated that measles transmission often exhibits strong geographic heterogeneity driven by variations in population density, vaccination coverage, mobility patterns, and socio-economic conditions (11–13). Spatio-temporal modeling has also been used to identify clusters of transmission, forecast outbreak risk, and guide targeted immunization strategies (13, 14). These approaches have proven valuable for understanding the drivers of measles persistence and for improving the effectiveness of surveillance and vaccination programs.

Despite these advances, limited evidence exists on the spatio-temporal dynamics of measles transmission in Ethiopia using high-resolution surveillance data. Although national surveillance reports document the overall burden of measles, few studies have systematically examined how transmission varies across geographic regions and over time, or how environmental, demographic, and socio-economic factors contribute to these patterns. Moreover, Ethiopia has experienced a marked resurgence of measles cases in recent years, with a substantial increase in reported outbreaks following 2021. This resurgence coincided with disruptions to health services, population displacement, and interruptions to routine immunization activities associated with the armed conflict in northern Ethiopia, particularly in the Tigray region. Such disruptions may have altered the epidemiology of measles by creating immunity gaps and changing patterns of disease transmission. However, the extent to which these changes have influenced the spatial and temporal distribution of measles across the country remains poorly understood. Addressing this gap is essential for identifying high-risk areas and informing more targeted measles control and elimination strategies.

This study therefore aims to explore the spatio-temporal patterns and determinants of measles incidence in Ethiopia using routine surveillance data from 2018 to 2024. Specifically, it seeks to characterize spatial clustering and hotspot persistence across regions and time, identify local risk factors associated with measles transmission using spatio-temporal modelling and explore temporal trends over the years. The findings will generate actionable evidence to support measles control and elimination efforts in Ethiopia and similar low-resource settings.

## Methods

### Study area and period

**Figure 1:**
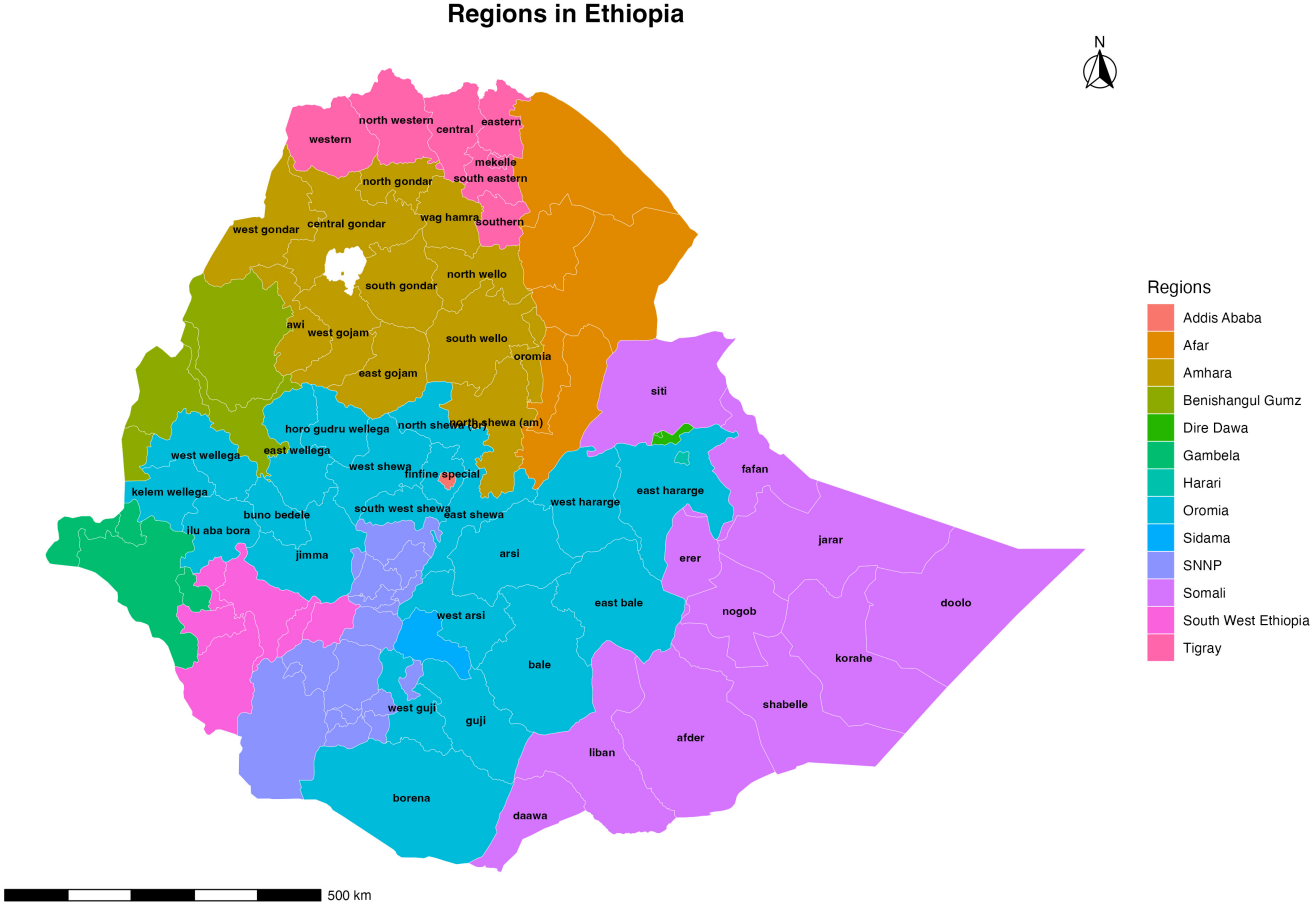
Administrative regions and zones of Ethiopia. Key regions and selected zones referenced in the analysis are annotated to facilitate geographic interpretation of the spatial results presented in subsequent figures.

The study was conducted across all the regions and city administrations and the zones within Ethiopia routinely reporting measles data to the Ethiopian Public Health Institute. To improve spatial interpretation, Figure 1 presents both regional and zonal administrative boundaries, with regional names annotated and selected zones highlighted for reference. The country exhibits substantial geographic and socio-demographic diversity, with large regional variations in population density, urbanization, transportation accessibility, and health service coverage. The world bank estimated total population of Ethiopia for 2025 to be 132,059,767 out of which 22.1% lived in urban areas while the remaining three quarter of the population lived in rural areas (15). Official reports indicate that the primary health service coverage has increased from 50.7% to over 90% since 1990 and currently the country has about 3,907 health centers, and 15,531 health posts delivering services to the population including routine immunization of Measles Containing Vaccine (MCV_1_ and MCV_2_) (16). We analyzed measles case-based data of 6 years (2018–2024) to assess the spatial and temporal distribution of cases and the associated determinants.

### Study design

The study employed a retrospective observational study utilizing both national and subnational (zonal level) surveillance data on measles and additional covariates including routine MCV_1_ coverage, rain fall, temperature, humidity, population density, distance to healthcare facility, relative wealth index, malnutrition indices such as wasted rate, stunted rate, underweight rate and time lagged and spatial lagged case indicators identified from various sources.

### Source and study population

All population living in the country during the study period (2018–2024) were considered the source population while all reported measles patients including laboratory confirmed, epi linked and clinically compatible cases were considered the study population.

### Sample size and sampling procedure

All reported measles cases during the period January 01, 2018–December 31, 2024 were included for analysis.

### Data collection and quality control

Measles is one of the 13 immediately reportable surveillance diseases in Ethiopia and these activities are coordinated by the Public Health Emergency Management (PHEM) under the Ethiopian Public Health Institute. All suspected measles cases are investigated by healthcare facilities and reported using a standard case reporting system. Confirmation is done through collecting and testing the patients' blood for the presence of measles immunoglobulin (IgM). All reported cases are then registered in the national measles surveillance registry. Variables such as unique ID number, age, sex, date of onset, place of residence, measles doses received, blood test results for IgM, date specimen collected, date specimen was received, date results were due, final classification, and outcome are recorded in the registry.

While the measles case-based surveillance data was obtained from the Ethiopian Public Health Institute, the remaining zonal level covariates were obtained from various sources. For the purpose of estimating zonal level malnutrition indicators such as wasting, stunting and underweight, we used Ethiopian DHS data from DHS Program. Climate variables including rainfall used in this study were downloaded from Climate Hazard Infrared Precipitation with Station data (CHIRPS). Zonal level access to healthcare facility was obtained from the Humanitarian Data Exchange (HDX). Population density and relative wealth index data were obtained from WorldPop. Vaccination data for MCV_1_ was downloaded from the Ethiopian Ministry of Health DHIS2 database. Night light used as a proxy indicator of economic activity and urbanization was acquired from Earth Observatory Group website at the University of Colorado.

Data quality issues including incompleteness of key variables were addressed using imputation and triangulations with other data sources. Specifically, we conducted imputation of missing or 0 age data using the median. Official reports of immunization coverage data were compared with that of DHS estimates to modify exaggerated figures. Additional data cleaning and management activities were also conducted before analyzing the data.

### Study variables

#### Outcome variable

This main dependent variable in this study was the reported count of measles cases per zone. Since the objective of the analysis was to identify spatio-temporal and environmental determinants of reported measles burden rather than to estimate population-standardized incidence rates, the models were specified using case counts. Population density was included as a covariate to partially account for differences in settlement patterns and population distribution across zones.

#### Independent variables

Various demographic, socioeconomic, epidemiological, environmental, and vaccination-related factors were used as independent variables in this study. Patient-related independent variables included age, region of origin, number of measles vaccine doses received, final case classification, and clinical outcome. Epidemiological variables such as date of onset of measles, date the specimen was received, and date results were dispatched were also included. Programmatic and sociodemographic covariates included population density, routine MCV_1_ vaccination coverage, night lights and relative wealth index. Environmental predictors such as rainfall, temperature, humidity, and prevalence of malnutrition (wasting, stunting, and underweight) were included to account for ecological and climatic influences on measles transmission. In addition, temporal and spatial dependence structures were captured by including lagged measles case counts (cases in the preceding biannual period) and spatially lagged cases (average number of cases in neighboring zones). These variables allowed the model to account for both within-zone persistence of transmission and spatial diffusion across adjacent areas, providing a more comprehensive representation of measles dynamics across space and time. Population density was included as a covariate to account for variation in settlement patterns across zones. Because reliable zonal population estimates matching the temporal resolution of the dataset were not available, a population offset term was not incorporated into the regression models.

### Data analysis

Data analysis was conducted using R statistical software version 4.5.1. Case-based measles surveillance data from 2018–2024 were merged with various zonal level socio-demographic, economic, epidemiological and environmental variables obtained from secondary sources. The variable for measles vaccination dose was recoded into four categories: 0 doses, 1–2 doses, >2 doses, and missing. Similarly, the measles IgM laboratory test result was classified as positive, negative, not done, pending or unknown.

Descriptive analyses summarized measles cases and deaths by zone, age group, vaccination status, IgM result, and year. Biannual trends maps were plotted to visualize temporal patterns, and summary statistics (mean, SD, median, IQR) were calculated for key covariates.

Spatial analysis assessed geographical clustering of measles cases using biannual zonal aggregates. Global Moran's I tested for overall spatial autocorrelation, while LISA and Getis–Ord Gi^*^ statistics identified hotspots, cold spots, and spatial outliers. Spatial analyses and mapping were performed using the spdep, sf, tmap, and spatialreg packages in R.

To identify determinants of measles occurrence, a multivariable negative binomial regression model was fitted to zonal-level case counts, including mid-year population to adjust for population size differences. Model results were presented as Incidence Rate Ratios (IRRs) and 95% confidence intervals. Covariates were considered statistically significant when the *p*-values were < 0.05. Model fit and checks for multicollinearity were evaluated using Akaike Information Criterion (AIC), Variance Inflation Factor (VIF), and Moran's I on residuals. Because measles incidence was measured repeatedly across 14 biannual periods, observations within the same time block were likely correlated due to shared epidemic cycles, vaccination campaign timing, and surveillance dynamics. To account for this non-independence and unobserved temporal heterogeneity, a random intercept for biannual periods was included in the negative binomial mixed model. Model comparison using Akaike Information Criterion (AIC) supported the inclusion of the random effect.

## Results

### Characteristics of the measles cases

Out of the total measles cases reported across all regions of Ethiopia between 2018 and 2024, the majority originated from Oromia (32.6%), followed by Amhara (12.7%) and Somali (10.2%) regions. Smaller proportions were reported from regions such as Harari, Dire Dawa, and Tigray, each contributing around 1% or less. The median age of reported cases was 48 months (IQR: 24–120), indicating that most affected individuals were young children. The number of reported measles cases increased substantially over time, peaking in 2023 (33.3%) and 2024 (41.6%), suggesting a recent resurgence of transmission. Most cases (67%) either didn't receive measles vaccine or had unknown vaccination status. The majority of specimens were classified as measles-positive (85%), and nearly all cases (99%) were reported as alive at the time of notification. Overall, the findings indicate that measles continues to affect multiple regions of Ethiopia, with a notable increase in recent years (Table 1).

**Table 1:** Summary of the characteristics of measles cases reported at the zonal level in Ethiopia between 2018 and 2024, (*n* = 71,635).

Characteristics		Frequency (%)
Age months: median (IQR)		48 (24–120)
Region	Afar	772 (1.1)
	Amhara	9,087 (12.7)
	Somali	7,297 (10.2)
	Harari	90 (0.13)
	Benishangul G.	1,867 (2.6)
	Oromia	23,382 (32.6)
	Tigray	743 (1.04)
	Dire Dawa	96 (0.13)
	Gambella	1,486 (2.1)
	Addis Ababa	1,367 (1.9)
	South Ethiopia	8,751 (12.2)
	S.W. Ethiopia	10,112 (14.1)
	Sidama	3,464 (4.8)
	Central Ethiopia	2,281 (3.2)
Year of onset	2018	1,597 (2.2)
	2019	3,992 (5.6)
	2020	1,942 (2.7)
	2021	1,933 (2.7)
	2022	8,553 (11.9)
	2023	23,830 (33.3)
	2024	29,788 (41.6)
Specimen received in days: median (IQR)	2 (4–6)	
Number of measles doses	0	28,265 (40)
	1–2	23,503 (31)
	>2	204 (2)
	Unknown	19,647 (27)
Specimen condition	No	1,672 (2)
	Adequate	9,692 (14)
	Not adequate	166(0.2)
	Missing	60,105 (84)
Measles Ig	Negative	3,341 (5)
	Positive	8,736 (12)
	Not done	516 (1)
	Pending	1,270 (2)
	Unknown	57,770 (81)
Final classification	Lab confirmed	8,736 (12)
	Epi-linked	61,100 (85)
	Clinically compatible	1,799 (3)
Outcome	Alive	60,765 (99)
	Dead	422 (1)
	Missing	10,448

### Description of environmental and health system related factors

The distribution of potential determinants of measles occurrence across Ethiopian zones shows considerable variation. Median rainfall was 12.39 mm (IQR: 5.87–25.28), while the average temperature was 20.74 °C (IQR: 18.85–23.74), reflecting the country's diverse climatic conditions. Median humidity was relatively low at 1.64 (IQR: 1.43–1.84). Population density varied widely, with a median of 148.36 people per km^2^ (IQR: 40.18–207.96), suggesting notable differences in population concentration across regions. The median distance to the nearest healthcare facility was 32.42 km (IQR: 17.54–65.56), indicating that access to health services remains a challenge for many communities. The relative wealth index had a negative median value of −0.33 (IQR: −0.43 to −0.29), suggesting generally low socioeconomic conditions including poverty (Table 2).

**Table 2:** Description of selected zonal level socio-economic, epidemiological, environmental, nutritional, and health system related factors affecting measles transmission.

Factors	Median (IQR)
Rain falls (mm)	12.39 (5.87–25.28)
Average temperature (°C)	20.74 (18.85–23.74)
Humidity	1.64 (1.43–1.84)
Population density	148.36 (40.18–207.96)
Distance to health care facility (KM)	32.42 (17.54–65.56)
Relative wealth index	−0.33 (−0.43–−0.29)
Vaccination coverage % (MCV_1_)	57.00 (46.00–68.00)
Wasted rate (malnutrition)	0.086 (0.051–0.141)
Stunted rate	0.31 (0.22–0.39)
Underweight rate	0.28 (0.21–0.36)
Night lights (economic activity)	0.0054 (0.0017–0.016)
Spatial lags	49.60 (38.44–78.51)
Lagged cases (temporal)	2.00 (0.00–16.00)

Zonal level vaccination coverage was relatively low, with a median of 57% (IQR: 46.00–68.00%). The median rates of wasting, stunting, and underweight were 8.6%, 31%, and 28%, respectively, highlighting a substantial burden of child malnutrition. The nightlight intensity, a proxy for economic activity and interaction among people, was very low (median 0.005), reflecting limited development in many areas. Finally, the spatial lag of measles cases had a median of 49.6 (IQR: 38.44–78.51), and lagged cases had a median of 2 (IQR: 0–16), indicating moderate spatial and temporal dependence in measles transmission (Table 2).

### Overall distribution of measles cases by zones in Ethiopia between 2018 and 2024

**Figure 2:**
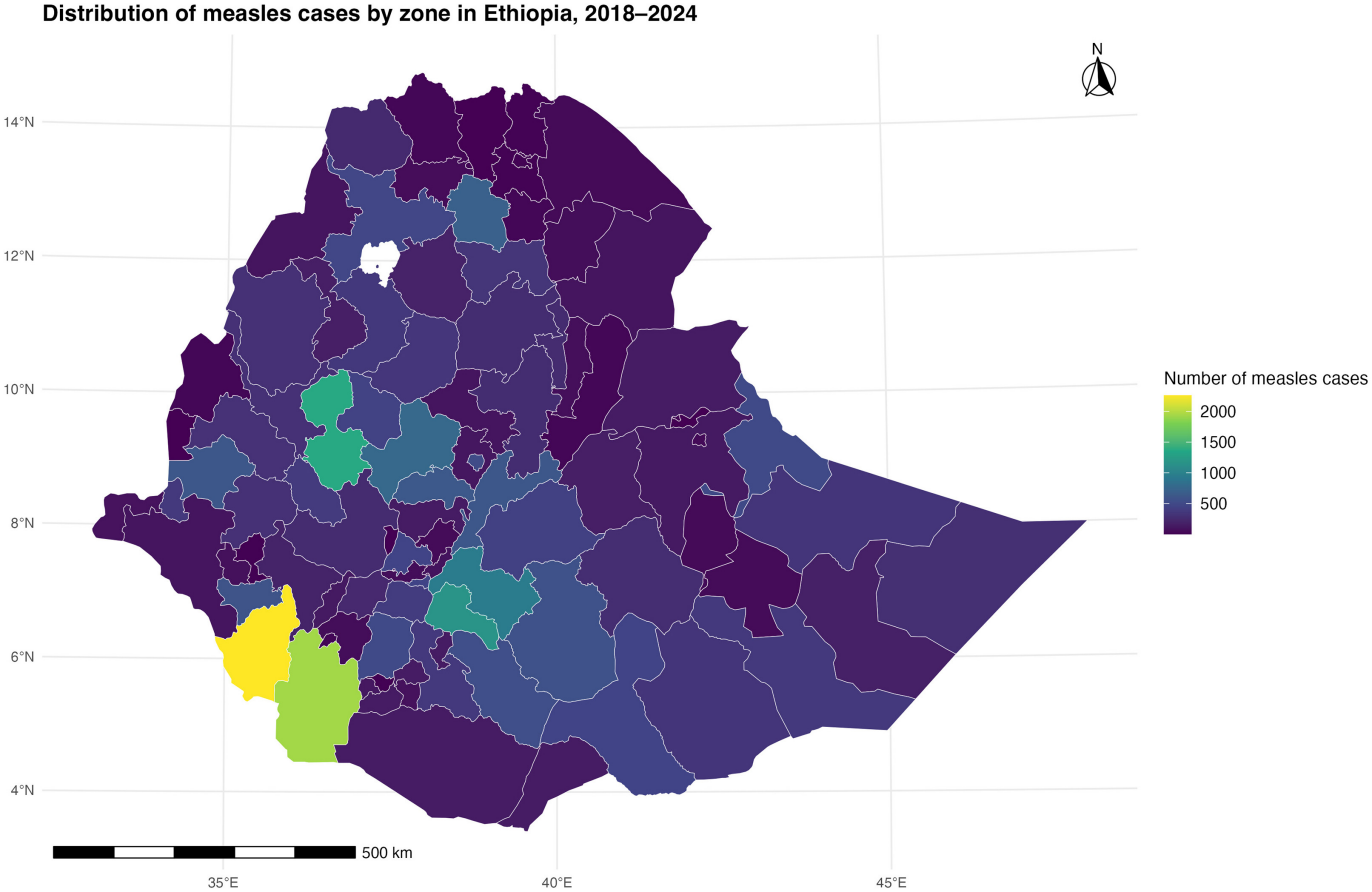
Spatial distribution of the number of measles cases by zone in Ethiopia (2018–2024).

The aggregate spatial distribution of measles cases from 2018 to 2024 reveals a pattern of extreme geographic concentration, driven by a few critical administrative zones. The vast majority of the national case burden is highly clustered in a small number of zones in the Southwestern part of Ethiopia (visualized as yellow and bright green on the map), accumulating the highest total case counts (>6,000). Additionally, zones in the southern and central parts of the country have substantial number of measles cases (>4000) while mostly fewer cases were observed in the both in South and North Eastern parts of the country (Figure 2). Conversely, the overwhelming majority of the country's zones (dark purple) reported minimal cumulative cases. This distribution confirms that measles is not a uniform national problem but a phenomenon of deeply rooted endemicity restricted to specific, geographically defined areas, demanding a highly localized and prioritized public health response.

### Zonal level temporal variation in the total number of measles cases in Ethiopia between 2018 and 2024

**Figure 3:**
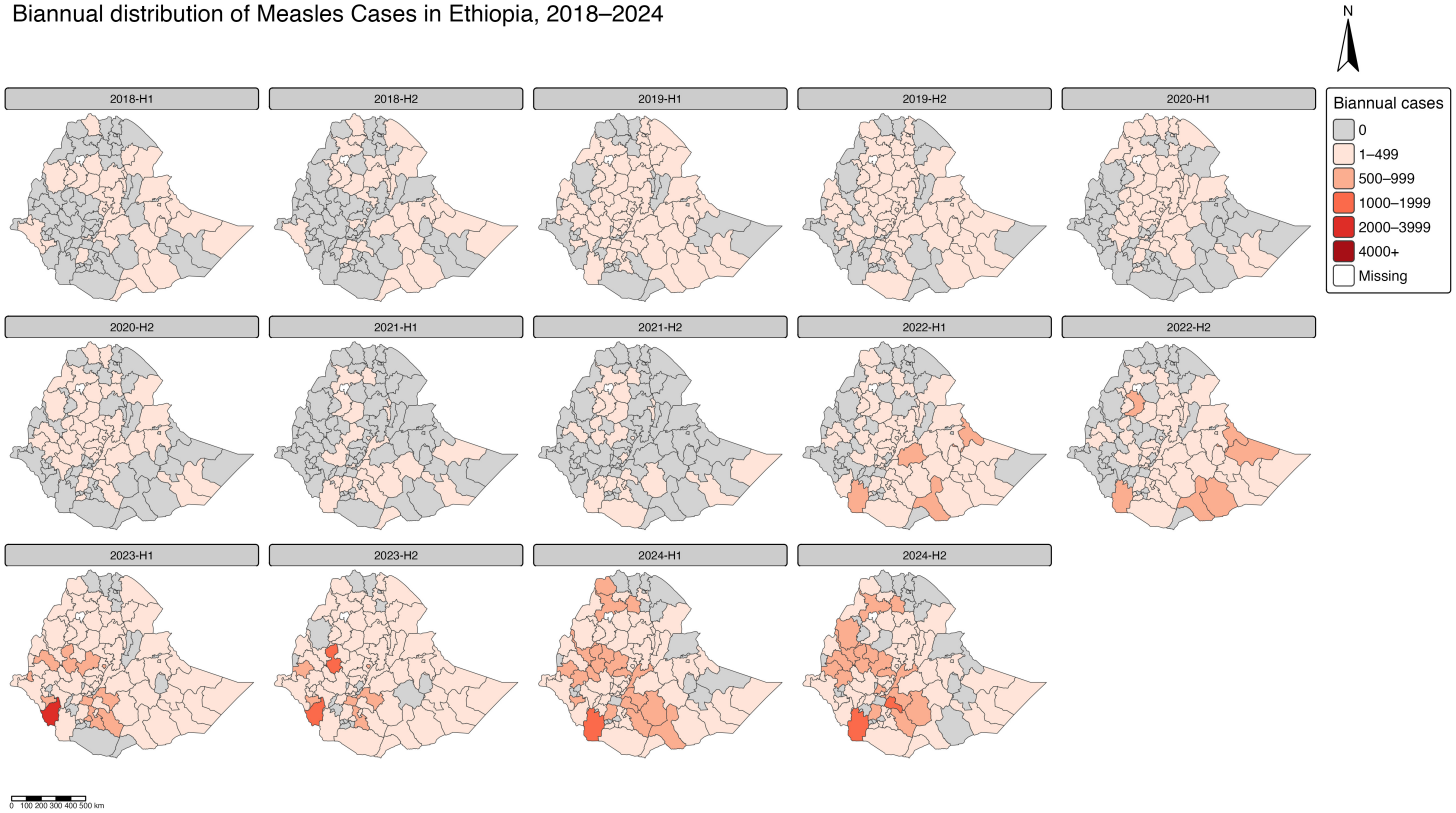
Bi-annual distribution of the total number of measles cases by zones in Ethiopia (2018–2024).

The map series illustrates the changing geographic and temporal distribution of reported measles cases in Ethiopia over 14 biannual periods from 2018 to 2024. Overall, the spatial pattern reveals substantial heterogeneity, with cases concentrated in a few high-burden regions particularly Oromia, Somali, and parts of the Southern and Central Ethiopia regions. Between 2018 and 2021, measles transmission remained relatively low and scattered, with limited outbreaks mainly in the eastern and southern zones. However, beginning in the second half of 2022, a sharp resurgence occurred, with a pronounced expansion of affected zones and a marked increase in case intensity that peaked during 2023–2024 (Figure 3). This escalation likely reflects a combination of accumulated immunity gaps following COVID-19 related service disruptions, suboptimal supplemental immunization activities, and high population mobility in endemic areas. By 2024, the distribution indicates partial containment in some regions but continued high caseloads in the southern and central zones, suggesting persistent foci of transmission and incomplete interruption of measles circulation.

### Global spatial distribution of measles cases at the zonal level in Ethiopia (2018–2024)

Global Moran's I analysis indicated weak but statistically significant positive spatial autocorrelation (Moran's I = 0.154, *p* = 0.003). This suggests that the distribution of measles cases at the zonal level in Ethiopia was heterogenous with areas of significant clustering as well as dispersion. This in turn means that zones with high numbers of measles cases were more likely to be located near other high-case zones, and low-case zones were clustered near other low-case zones, rather than being randomly distributed.

### Local Indicators of Spatial Association (LISA)

**Figure 4:**
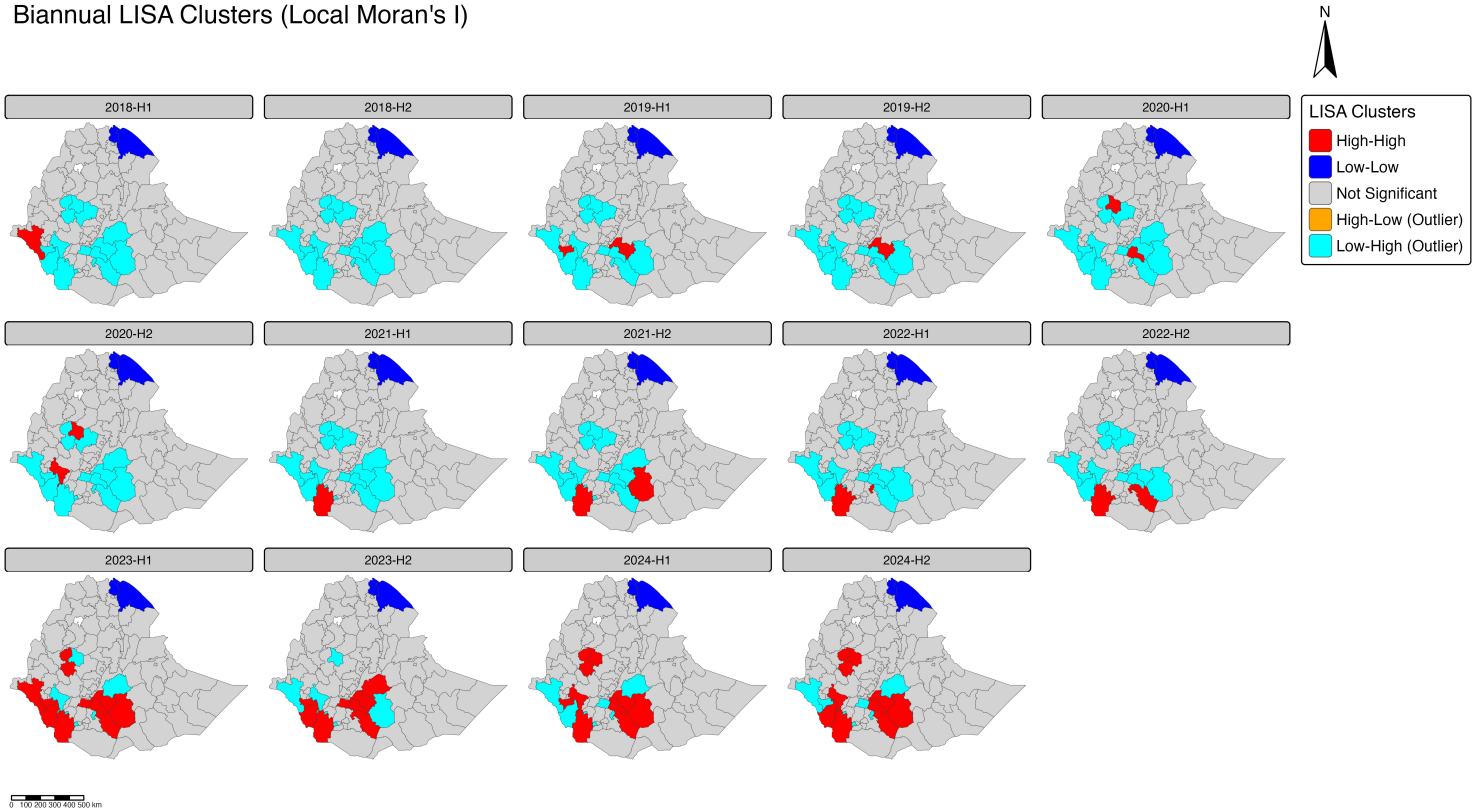
Biannual map for zonal level Local Indicators of Spatial Association of measles cases in Ethiopia, 2018–2024.

The map below displays the LISA outliers at the zonal administrative level in Ethiopia across the 2018–2024 period, specifically focusing on identifying localized spatial anomalies. This analysis isolates zones whose measured variable value is significantly different from that of their immediate neighbors. High-Low (HL) outliers (orange) pinpoint isolated “hotspots” where a zone's high value is surrounded by low-value neighbors. Conversely, Low-High (LH) outliers (cyan) highlight “coldspots” where a zone with a low value persists despite high-value surroundings. Zones colored gray indicate non-significant patterns while zones colored in red and blue represent High-High hotspots and Low-Low cold spots respectively. Understanding the unique status of these critical spatial deviations could help in outbreak response efforts (Figure 4).

The analysis revealed spatial heterogeneity in measles incidence across Ethiopia. While High-High clusters (hotspots, red) emerged in southern and central regions and Low-Low clusters (coldspots, blue) persisted in northern and eastern regions, notable Low-High outliers (cyan) highlighted localized areas of fewer cases relative to their neighbors. These outliers were most prominent in early periods (2018–2021), particularly in parts of Oromia, Southern and South Western Ethiopia, often along the edges of higher-incidence zones (Figure 3). Their occurrence suggests pockets of lower transmission within generally high-incidence neighborhoods. However, no specific area of high-low outlier was observed in this analysis.

### Static hotspots of measles cases in Ethiopia between 2018 and 2024

The Getis-Ord statistic calculates a Z-score for every zone, determining if the case count in that zone and its neighbors is significantly higher or lower than the national average. The map reveals a highly polarized distribution of the measles burden.

**Figure 5:**
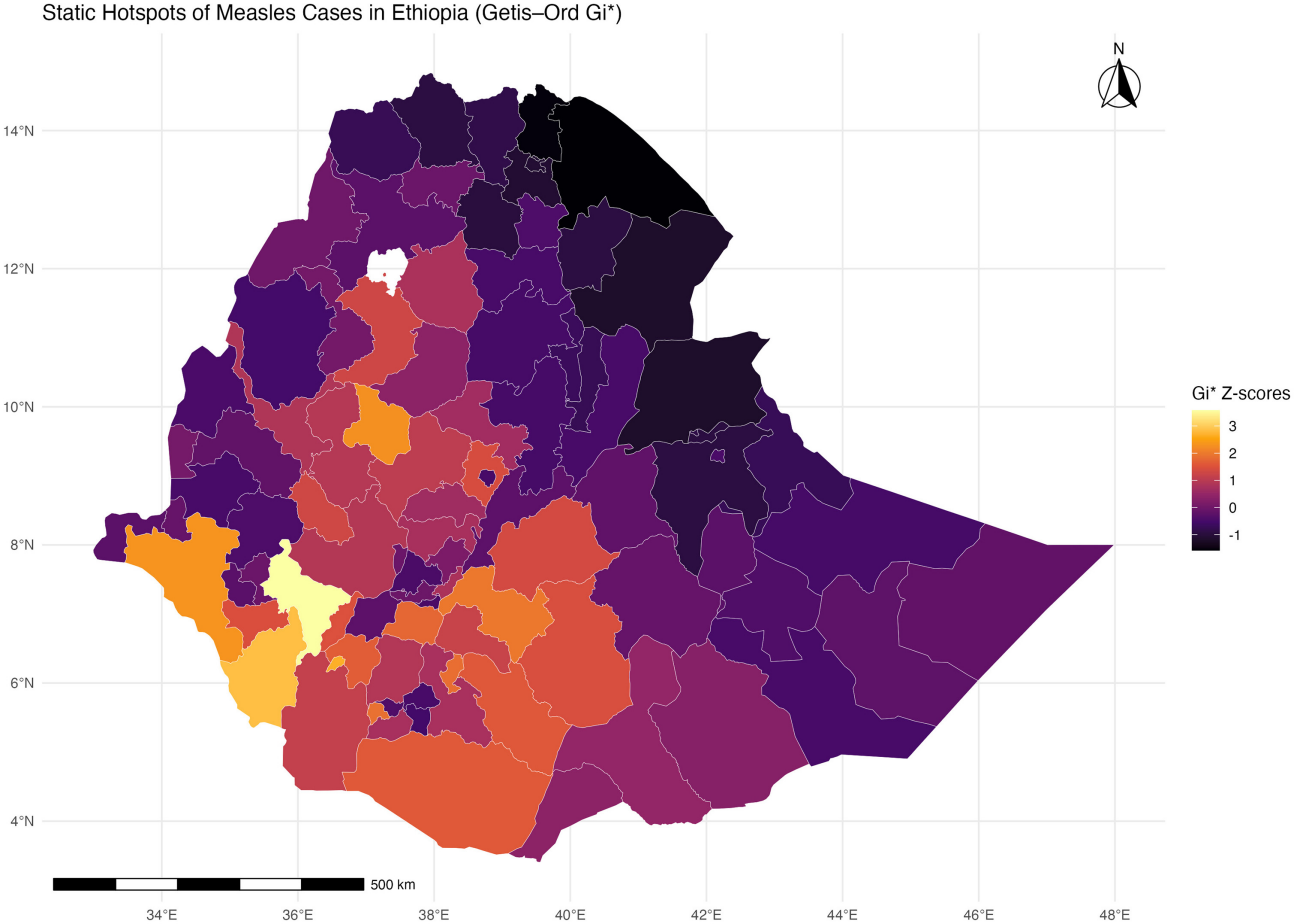
Zonal level static hotspots of measles in Ethiopia (2018–2024).

**Figure 6:**
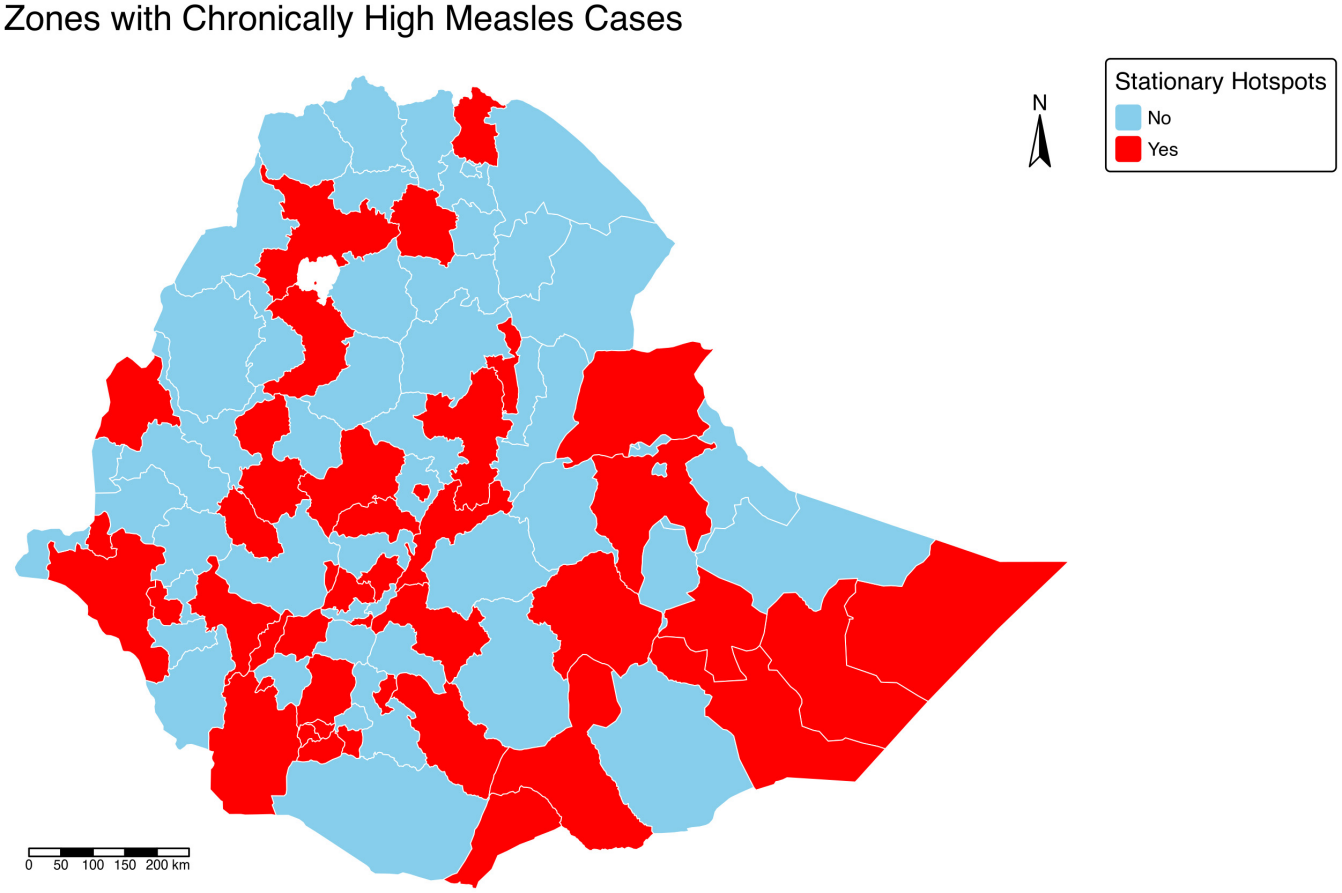
Map of zones with stationary hotspots of measles cases (> 50% of the weeks) in Ethiopia, 2018–2024.

The spatial hotspot analysis of measles cases in Ethiopia between 2018 and 2024 again revealed marked geographic heterogeneity in disease clustering across zones. High and statistically significant hotspots (zones with high Gi^*^ Z-scores) were primarily concentrated in the southern and western parts of the country. Specifically, zones within the Southern and South Western Ethiopia regions, including Wolayita, Gamo, Gofa, South Omo, and Bench Sheko, as well as Kaffa and Sheka zones, exhibited persistent clustering of high measles incidence. Similarly, strong hotspots were observed in parts of Oromia Region, particularly Guji, West Guji, Borena, and West Arsi zones, extending toward the southern border areas (Figure 5). Furthermore, zones with long standing or stationary hotspots i.e., areas or zones where hotspots have persisted for more than 50 weeks throughout the study period have been identified (Figure 6). These areas could possibly correspond to regions with historically low vaccination coverage, high population movement, and limited healthcare accessibility, suggesting that underlying programmatic and socioeconomic factors may contribute to sustained transmission.

In contrast, coldspots zones characterized by significantly low measles incidence were predominantly located in the northern and eastern parts of Ethiopia. Most notably, Tigray Region, the Afar lowlands, and parts of Somali Region showed consistent cold spot patterns, indicating relatively lower reported measles case counts during the study period. These areas may have benefited from more effective outbreak control interventions, better campaign reach in earlier years, or under-reporting due to limited surveillance or nomadic population movements. The central highlands, including Addis Ababa and nearby zones such as North Shewa and East Gojam, generally exhibited intermediate Gi^*^ Z-scores, reflecting moderate or fluctuating transmission patterns without consistent clustering.

The spatial distribution indicates a clear south–north gradient in measles clustering, with the southern and southwestern zones forming persistent high-risk foci. The identification of these zones as hotspots provides critical evidence for prioritizing targeted immunization campaigns, enhanced active surveillance, and microplanning for outbreak response.

### Time varying hotspots of measles cases by zone in Ethiopia (2018–2024)

**Figure 7:**
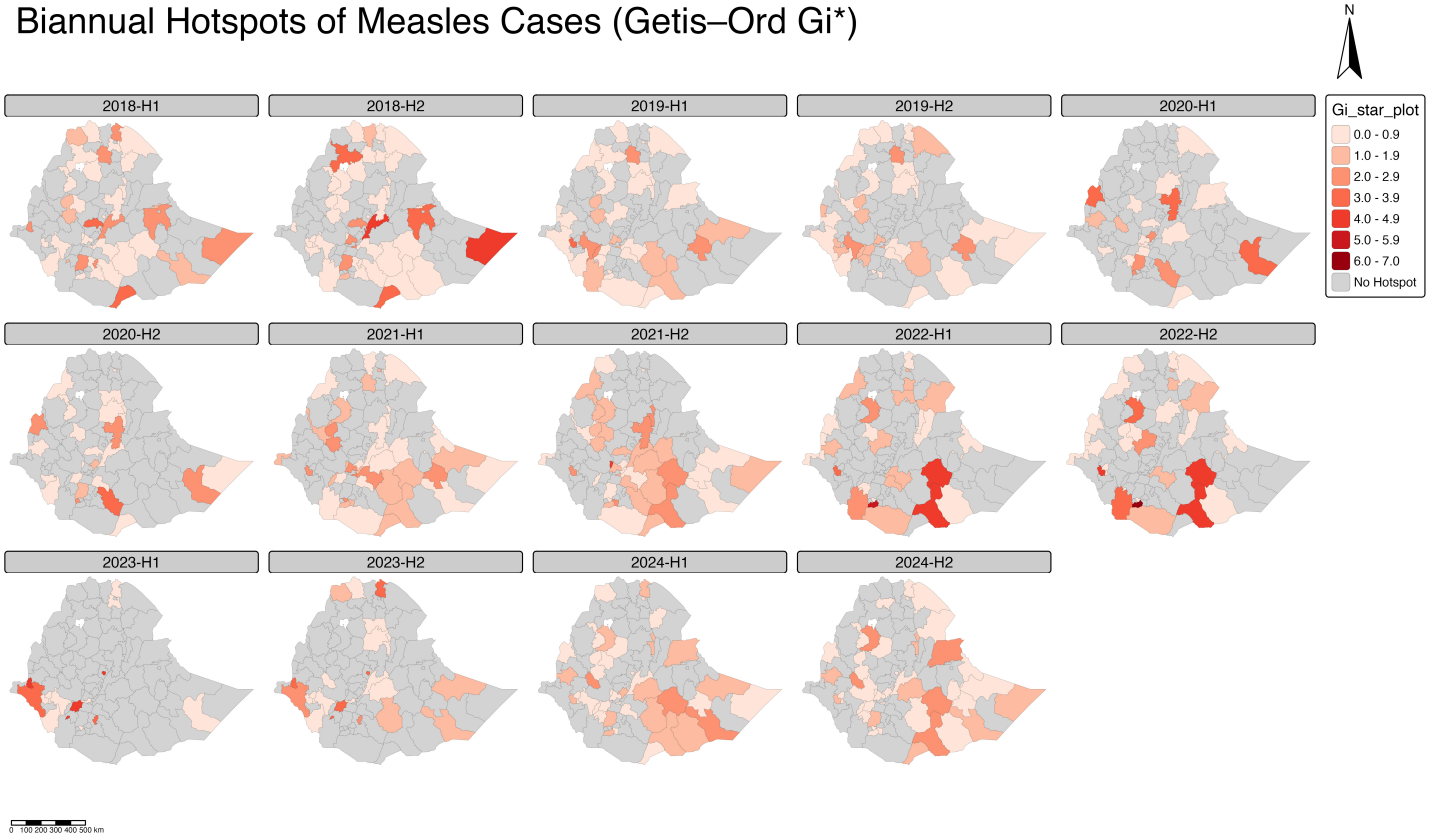
Time varying (bi-annual) map of the hotspots of measles cases by zone in Ethiopia between 2018 and 2024.

The biannual spatiotemporal hotspot analysis of measles cases in Ethiopia from 2018 to 2024 revealed dynamic clustering patterns that shifted both spatially and temporally across the study period. During the early years (2018–2020), hotspots were relatively limited but consistently appeared in the South and South Western Regions particularly in Wolayita, Gamo, and South Omo zones and in parts of Oromia, including Guji and West Arsi. These zones showed repeated clustering across multiple half-year intervals, indicating sustained transmission chains likely linked to suboptimal immunization coverage and population mobility. Coldspots, on the other hand, were more pronounced in northern Ethiopia, including Tigray, Afar, and Amhara highlands, where lower Gi^*^ values reflect reduced measles transmission during the same periods (Figure 7).

From 2021 to 2023, the pattern intensified, with an expansion of high Gi^*^ clusters in the southwestern parts, encompassing Bench Sheko, Kaffa, and Sheka zones, as well as re-emergent hotspots in Borena and West Guji. This temporal concentration of hotspots suggests repeated outbreaks in regions with weak routine immunization systems and delayed outbreak response mechanisms. The presence of multiple contiguous high-value Gi^*^ zones during these intervals' points to possible spatial diffusion and localized epidemic persistence, rather than isolated case occurrences. Meanwhile, the central highlands, including zones around Addis Ababa and Oromia's central belt, generally remained neutral or cold, reflecting more stable measles control and likely better health system coverage (Figure 7).

By 2024, the distribution of hotspots showed a resurgence in the southern border areas and a few newly emerging clusters in western Oromia and Gambella, implying shifting transmission dynamics influenced by cross-border movement, or changing environmental conditions (Figure 7).

### Spatio-temporal modeling of measles cases in Ethiopia

A total of 13 covariates were included to model the measles case counts of over 6 years in each zone of Ethiopia. These covariates included socio-economic, environmental, nutrition, health service coverage and epidemiological factors. Rainfall, humidity, stunted rate, and wasted rate were excluded from the final model due to multicollinearity, as indicated by high variance inflation factors (VIF > 6) and strong pairwise correlations with other covariates. Other variables such as vaccination coverage and population density were excluded during model development as they very had high *p*-values. This was done through stepwise back ward regression where the covariates with the highest *p*-values are successively removed. Variables having a *p*-value less than 0.05 were considered statistically significant.

Both Poisson regression and negative binomial regression models were fitted to the observed data. The Poisson model demonstrated substantial overdispersion, whereas the negative binomial model showed improved model fit with a lower AIC and an acceptable dispersion parameter (0.235). Therefore, the final model was specified using the negative binomial regression framework.

### Factors associated with the distribution of measles cases across zones in Ethiopia (2018–2024)

The spatio-temporal negative binomial model identified several demographics, climatic, and socioeconomic factors that significantly shaped measles incidence across Ethiopian zones between 2018 and 2024. Both temporal and spatial dependence were strong predictors of transmission. The temporal lag of measles cases (IRR = 1.24, 95% CI: 1.02–1.51, *p* = 0.028) indicated that higher case counts in preceding periods increased current incidence, reflecting persistence between epidemic waves. Spatial dependence was even stronger: zones neighboring high-incidence areas experienced a significantly elevated risk (IRR = 1.73, 95% CI: 1.47–2.03, *p* < 0.001), consistent with the pronounced hotspot patterns observed in Oromia, Somali, South Ethiopia, and parts of Amhara and Afar (Table 3, Supplementary figure 1).

**Table 3:** Summary of the spatio-temporal regression model to assess factors associated with measles distribution in Ethiopia (2018–2024).

Factors	IRR	Std. error	Z-score	*P*-value	95% CI
Intercept	19.9	0.418	7.150	8.67e-13 ^***^	[8.77–45.2]
Temporal lags	1.24	0.098	2.193	0.02828 ^*^	[1.02–1.51]
Spatial lag cases	1.73	0.081	6.724	1.77e-11 ^***^	[1.47–2.03]
Average temperature	0.78	0.087	−2.822	0.00478 ^**^	[0.66–0.93]
Underweight rate	1.32	0.532	0.519	0.60393	[0.46–3.74]
Night lights	2.21	0.150	5.277	1.31e-07 ^***^	[1.64–2.96]
Relative Wealth Index	0.40	0.179	−5.093	3.52e-07 ^***^	[0.27–0.56]
Distance to health facility	0.95	0.06794	−0.805	0.420684	[0.28–1.27]

^*^Significant at *p* < 0.05; ^**^ significant *p* < 0.01; ^***^significant *p* < 0.001.

Climatic conditions also contributed to spatial variation. Higher average temperatures were associated with lower measles incidence (IRR = 0.78, 95% CI: 0.66–0.93, *p* = 0.005), suggesting reduced viral survival or transmission efficiency in hotter, drier lowland zones. Higher prevalence of underweight children was positively associated with measles incidence (IRR = 1.32, *p* = 0.604), although the association was not statistically significant (Table 3).

Socioeconomic factors were found to have significant association with zonal level measles distribution. Higher scores on the relative wealth index substantially reduced measles risk (IRR = 0.40, 95% CI: 0.27–0.56, *p* < 0.001), indicating that poorer zones remain disproportionately affected. Night-light intensity showed a strong positive association with measles transmission (IRR = 2.21, 95% CI: 1.64–2.96, *p* < 0.001). Brighter night lights serving as proxies for higher population density and economic activity were linked to increased measles counts, suggesting that areas with greater human concentration and mobility experience higher contact rates and therefore greater transmission. Distance to health facilities was not a significant predictor after adjusting for the other covariates (Table 3, Supplementary figure 1).

The inclusion of a random intercept for biannual periods significantly improved model fit (SD = 1.45), suggesting considerable temporal variability in measles incidence across the study period even after adjusting for all the covariates. This variability likely reflects fluctuations in national outbreak dynamics, supplementary immunization activities (SIAs), reporting rates and other seasonal effects that were not fully captured by climatic covariates.

## Discussion

This study provides a comprehensive spatio-temporal analysis of measles incidence in Ethiopia from 2018 to 2024, revealing substantial regional heterogeneity and highlighting the multifactorial determinants shaping measles transmission. Our findings demonstrate that measles incidence was not uniform across the country but rather concentrated in specific geographic zones, particularly in southern, southwestern, and parts of central Ethiopia, including Oromia, Somali, South Ethiopia, and Amhara regions. Conversely, regions such as Harari, Dire Dawa, Tigray, and Gambella exhibited relatively low burdens. Temporally, measles cases increased substantially after 2020, peaking in 2023–2024, likely reflecting post-COVID-19 disruptions in routine immunization, immunity gaps, and improved outbreak detection activities. These trends are consistent with global evidence indicating resurgence of vaccine-preventable diseases following pandemic-related service interruptions, particularly in low- and middle-income countries (17–19).

Our spatial analyses provide further insights into the distribution and persistence of measles. Moran's I test confirmed significant positive spatial autocorrelation (Moran's I = 0.154, Z = 2.75, *p* = 0.003), indicating that high-incidence zones tend to cluster together while low-incidence zones are similarly grouped, rather than being randomly distributed (18). LISA identified high-high clusters (hotspots) predominantly in southern and central Ethiopia and low-low clusters (coldspots) in northern and eastern regions. LISA also highlighted high-low and low-high outliers, revealing localized elevations or reductions in incidence relative to neighboring zones. The observed spatial heterogeneity in measles incidence may reflect differences in vaccination coverage, accessibility to health services, and socio-economic conditions across zones, as indicated by the associations observed with MCV1 coverage, distance to health facilities, and wealth-related indicators in our analysis (20, 21).

Complementing this, the Getis-Ord Gi^*^ hotspot analysis demonstrated persistent and stationary hotspots in southern and southwestern Ethiopia, including Wolayita, Gamo, Gofa, South Omo, Bench Sheko, Kaffa, and Sheka, as well as parts of Oromia such as Guji, West Guji, Borena, and West Arsi. These zones exhibited significantly higher case counts over more than 50% of the weeks studied, indicating long-standing vulnerabilities likely linked to weak routine immunization, high population mobility, and limited healthcare access (18, 19). Coldspots were concentrated in Tigray, Afar lowlands, and parts of Somali, suggesting either effective control measures, lower population density, or potential under-reporting in nomadic communities. The integration of spatial analyses with the negative binomial spatio-temporal model underscores the importance of geographic dependence, with strong spatial lag (IRR = 1.73, 95% CI: 1.47–2.03, *p* < 0.001) reinforcing the observed clustering of cases across neighboring zones. Temporal persistence (temporal lag IRR = 1.24, 95% CI: 1.02–1.51, *p* = 0.028) further highlights sustained transmission between epidemic waves, consistent with the role of human mobility in propagating outbreaks across adjacent zones (20, 21).

Climatic factors played a significant role in modulating measles transmission. Higher average temperatures were associated with lower incidence (IRR = 0.78, 95% CI: 0.66–0.93, *p* = 0.005), consistent with reduced viral survival or transmission efficiency in hotter, drier lowland zones (22–24). Although the underweight rate showed a positive association with measles incidence (IRR = 1.32), it was not statistically significant after adjustment for other covariates, suggesting that malnutrition may contribute to susceptibility but is less predictive at the ecological (zonal) level (25). Socioeconomic factors were strong predictors: higher relative wealth index was protective (IRR = 0.40, 95% CI: 0.27–0.56, *p* < 0.001), indicating that poorer zones remain disproportionately affected, likely due to poverty, lower immunization coverage, limited outbreak response capacity, and structural barriers to healthcare access (21, 25, 26). Night-light intensity, a proxy for economic activity and human concentration, was positively associated with transmission (IRR = 2.21, 95% CI: 1.64–2.96, *p* < 0.001), suggesting that zones with higher human mobility and congregation experience elevated contact rates and measles spread (18, 20).

Unlike earlier analyses, population density and distance to health facilities were not significant predictors after adjusting for other covariates, indicating that economic activity (night lights) and wealth disparities better capture the drivers of local transmission. These findings highlight the importance of targeting interventions based on actual spatial risk and socioeconomic vulnerability rather than crude population measures alone. The inclusion of a random intercept for biannual periods (SD = 1.45) further captured substantial temporal variability, reflecting epidemic cycles, variations in supplementary immunization activities, and year-to-year fluctuations in surveillance and outbreak dynamics (18, 19).

The combined evidence from spatio-temporal modeling and spatial clustering analyses emphasizes the need for geographically targeted interventions. Persistent hotspots and high-high LISA clusters indicate long-standing foci of transmission, which likely sustain measles outbreaks despite broader national campaigns. Conversely, coldspots and low-incidence outliers may inform regions where resources can be redirected to higher-risk zones. Integrating spatially explicit surveillance with routine immunization planning, micro-targeted outbreak response, and data-driven risk stratification can thus enhance the efficiency and effectiveness of measles control efforts (18–21).

The analytical framework applied in this study combining spatial statistics, temporal lag structures, and regression-based modeling may also be useful for investigating the transmission dynamics of other infectious diseases characterized by spatial and temporal heterogeneity. Integrative spatial and temporal modeling approaches that combine population data, risk factor assessment, and surveillance systems are increasingly used to better understand the distribution and determinants of infectious diseases such as COVID 19 (27), HBV (28). Comprehensive epidemiological investigations have also been proposed to examine HPV prevalence, risk factors, and screening strategies in different populations (29). Although the specific analytical techniques may differ depending on disease characteristics and available data, such integrative approaches can provide valuable insights for designing targeted public health interventions and strengthening disease control strategies.

This study has several strengths, including the use of a large multi-year dataset covering all Ethiopian zones, integration of robust spatio-temporal modeling with multiple spatial analyses (Moran's I, LISA, Getis-Ord Gi^*^), and consideration of climatic, nutritional, and socioeconomic covariates. Limitations include potential poor surveillance and under-reporting in conflict-affected or nomadic areas, incomplete laboratory confirmation, reliance on epidemiological linkage, and the ecological nature of the analysis, which limits inference to individual-level risk. Additionally, unmeasured confounders such as timing of campaigns, cold chain performance, and post-COVID-19 service disruptions may have influenced the observed associations. Because detailed zonal population estimates matching the temporal resolution of the surveillance data were not available, a population offset term was not incorporated in the regression models. Nonetheless, the findings from this study could contribute a great deal in the national effort toward measles elimination goal.

## Conclusion

This study demonstrates that measles incidence in Ethiopia between 2018 and 2024 was strongly shaped by spatial clustering, temporal persistence, climatic variability, and socioeconomic disparities. Persistent hotspots in southern and southwestern zones, coupled with lower wealth and higher human mobility, indicate that national-level coverage targets alone are insufficient for elimination. Achieving measles control will require geographically fine-tuned immunization strategies, integration of spatial modeling into routine surveillance, targeted interventions in high-risk zones, and the use of climatic and mobility data for early warning and micro-planning. Reducing spatial clustering of susceptibility, rather than solely increasing overall coverage, is likely critical to interrupting transmission in endemic settings.

Integrating spatio-temporal modeling with routine surveillance can enhance early detection and precision of outbreak response. Strengthening immunization services in marginalized and high-risk zones, improving child nutrition, and incorporating environmental and socioeconomic data into health planning are essential for sustained measles control and the eventual achievement of elimination goals.

## Glossary

EPHI: Ethiopian Public Health Institute; GIS: Geographic Information System; HH: High-High; HL: High-Low; ID: Identification; IgM: Measles Immunoglobulin; IQR: Interquartile Range; IRR: Incidence Rate Ratio; LISA: Local Indicators of Spatial Association; LH: Low-High; LL: Low-Low; MCV: Measles Containing Vaccine; PHEM: Public Health Emergency Management; RWI: Relative Wealth Index; SD: Standard Deviation; SIA: Supplementation Immunization Activity; VIF: Variance Inflation Factor; WHO: World Health organization

## Data Availability

The raw data supporting the conclusions of this article will be made available by the authors, without undue reservation.
