# Supplementary material for: Spatio-temporal patterns and determinants of measles incidence in Ethiopia between 2018 and 2024
Source: Front Public Health. 2026 Apr 9;14:1760450. doi: 10.3389/fpubh.2026.1760450 (PMC13102658; doi:10.3389/fpubh.2026.1760450)
Supplement: Supplementary file 1 [file Supplementary_file_1.docx]

**Supplemental material**

Supplementary figure S1

Figure S1: Adjusted incidence rate ratios from the final model

The forest plot (Supplementary Figure S1) summarizes the adjusted incidence rate ratios (IRRs) and corresponding 95% confidence intervals from the final negative binomial regression model. Lagged measles incidence was positively associated with current measles cases, indicating temporal persistence in transmission dynamics. Similarly, the spatial lag of cases showed a positive association, suggesting that measles incidence in neighboring districts contributes to increased risk in a given district, consistent with spatial spillover effects.

Among the covariates, higher levels of night-time lights were associated with increased measles incidence, potentially reflecting higher population density or greater urban connectivity that may facilitate transmission. In contrast, the relative wealth index showed a negative association with measles incidence, suggesting that districts with higher socioeconomic status may experience lower disease burden. Average temperature also showed a modest negative association with measles incidence.

Other variables, including underweight prevalence and distance to the nearest health facility, had confidence intervals that crossed the null value (IRR = 1), indicating that their associations with measles incidence were not statistically significant in the adjusted model. Overall, the forest plot visually confirms the direction and magnitude of the associations reported in the regression table presented in the main manuscript.
